# Intake of Unprocessed and Processed Meat and the Association with Cardiovascular Disease: An Overview of Systematic Reviews

**DOI:** 10.3390/nu13103303

**Published:** 2021-09-22

**Authors:** Marianne Uhre Jakobsen, Anette Bysted, Heddie Mejborn, Anders Stockmarr, Ellen Trolle

**Affiliations:** 1 Division for Diet, Disease Prevention and Toxicology, National Food Institute, Technical University of Denmark, 2800 Kgs. Lyngby, Denmark; 2 Division for Food Technology, National Food Institute, Technical University of Denmark, 2800 Kgs. Lyngby, Denmark; anby@food.dtu.dk (A.B.); hmej@food.dtu.dk (H.M.); eltr@food.dtu.dk (E.T.); 3 Department of Applied Mathematics and Computer Science, Technical University of Denmark, 2800 Kgs. Lyngby, Denmark; anst@dtu.dk

**Keywords:** cardiovascular disease, diet, poultry, red meat, systematic overview

## Abstract

We conducted an overview of systematic reviews to summarize reviews of cohort studies on intake of unprocessed and processed meat and the risk of cardiovascular disease (CVD), coronary heart disease (CHD), and stroke. Systematic reviews of cohort studies published between January 2010 and August 2020 were identified through a systematic literature search in PubMed, Embase, and Web of Science. The quality of how each review was conducted was assessed and the overall confidence in the results of each review was rated using AMSTAR 2. The quality of evidence of each meta-analysis was graded using NutriGrade. Three reviews were included, with meta-analyses of unprocessed red meat and CVD (*n* = 1) and stroke (*n* = 2); unprocessed poultry and stroke (*n* = 1); and processed meat and CVD (*n* = 1), CHD (*n* = 1), and stroke (*n* = 3). The overall confidence in the results of each review was rated as critically low. The meta-evidence was graded moderate for a positive association between unprocessed red meat and stroke and moderate for a positive association between processed meat and CHD and stroke. For other associations the meta-evidence was graded as low or very low. In conclusion, the associations between unprocessed and processed meat with CVD and major subtypes of CVD have not been extensively investigated.

## 1. Introduction

The American Meat Science Association defines ‘meat’ as: ‘skeletal muscle and its associated tissues derived from mammalian, avian, reptilian, amphibian, and aquatic species harvested for human consumption...’ [1], yet the term ‘meat’ is often equated to skeletal muscle and its associated tissue derived from mammalian species (also known as red meat) by nutrition researchers [2]. Certain meat cuts are converted industrially into a variety of different meat products by a host of technologies including salting, curing, fermentation, smoking, drying, or other processes to enhance flavour or improve preservation [1].

Red meat, most commonly associated with beef, pork, and lamb, is one of the main sources of nutrients and energy in the diet in Western countries. For example, in the Danish diet, red meat contributes 12% of the total energy intake [3]. Globally, coronary heart disease (CHD) and stroke are among the leading causes of disease burden [4], and diet is an important modifiable risk factor [5]. Red meat contains saturated fatty acids and haem iron, which both have been adversely associated with cardiovascular disease (CVD) risk [6,7,8]. Furthermore, processed meat may contain high amounts of sodium, which has also been linked with a higher risk of CVD [8]. The associations between the intake of unprocessed and processed meat with the risk of CVD and major subtypes of CVD have been investigated in a number of observational epidemiological studies and the evidence summarized in reviews. By systematically assessing primary research, systematic reviews aim to provide an up-to-date summary of the body of evidence [9]. The systematic reviews address a need by public health policy decision-makers to be able to access up-to-date information [9]. Dietary guidelines are based predominantly on findings from systematic reviews and meta-analyses of epidemiological observational studies on diet-disease relationships and mostly cohort studies [10]. This is because randomised controlled trials are not available or not considered applicable [10]. It is important that users of systematic reviews can identify high quality reviews. This underscores the need for assessment of the way in which a review is conducted.

The rapidly increasing number of systematic reviews have led to another form of evidence synthesis, namely overviews of systematic reviews (also known as umbrella reviews) [11]. Overviews are often broader in scope than any individual systematic review. They search for and identify multiple systematic reviews on related research questions in the same topic area for the purpose of summarizing systematic review evidence [11].

The aim of this study was to summarize systematic reviews of cohort studies on the intake of unprocessed and processed meat and the risk of CVD and major subtypes of CVD. We assessed the quality of how each review was conducted and rated the overall confidence in the results of each review using A MeaSurement Tool to Assess systematic Reviews (AMSTAR) 2 [12]. The quality of evidence of meta-analyses contained within the systematic reviews was assessed and graded using NutriGrade [13].

## 2. Materials and Methods

We conducted an overview of systematic reviews of cohort studies on the intake of unprocessed and processed meat and the risk of CVD and major subtypes of CVD. Our protocol, including review question, eligibility criteria, search strategy, and methods of quality assessment, was registered in PROSPERO (http://www.crd.york.ac.uk/PROSPERO, identifier CRD42020205880) prior to conducting the overview. We conducted the overview in accordance with methodological guidance for conduct of overviews of systematic reviews [11,14].

### 2.1. Study Elgibility Criteria

Studies were included if they met the following criteria: (1) it was a systematic review of cohort studies with or without meta-analysis and published between January 2010 and August 2020; (2) participants were recruited from the general adult population; (3) it considered at least one of the following exposures: intake of unprocessed red meat, intake of unprocessed poultry, or intake of processed meat; and (4) it considered at least one of the following outcomes: incidence of CVD, CHD, or stroke. A review was considered systematic if the review attempted to identify all the evidence meeting specified eligibility criteria (i.e., PECOS items) to answer the review question. There were no restrictions to language.

### 2.2. Literature Search

Studies were identified through a systematic literature search in the bibliographic databases PubMed, Embase, and Web of Science. The search included only terms related to exposures and outcomes. The full literature search strategy for PubMed is shown in Supplementary Materials Methods S1. We also screened the reference lists of included systematic reviews.

### 2.3. Study Selection

An eligibility assessment was performed independently by two reviewers (M.U.J. and A.B. or M.U.J. and E.T.) using Rayyan [15]. Titles and/or abstracts of records identified through the literature search were screened against the eligibility criteria. The full text of studies that appeared to meet the study eligibility criteria, or where there was any uncertainty about eligibility, was retrieved and assessed for eligibility. Any disagreement was resolved by discussion. The Preferred Reporting Items for Systematic Reviews and Meta-analyses (PRISMA) [16] flow diagram was used to summarize the study selection process.

### 2.4. Data Extraction

A data extraction sheet (developed a priori by the reviewers and pilot-tested) was used to extract data. For each study (referred to hereinafter as systematic review), we extracted the following data, if reported: first author’s last name, publication year, databases searched, date ranges of databases searched, exposure, definition of exposure, outcome, authors’ conclusion, and information on potential sources of conflict of interest. For each cohort study (referred to hereinafter as primary study) contained within each systematic review, we extracted first author’s last name, publication year, cohort name, study origin, exposure, outcome, gender, and risk of bias assessment, if reported. For each primary study meta-analysis included in the systematic reviews, we extracted exposure, outcome, type of comparison (high versus low or dose-response), number of primary studies, number of participants, number of events, summary risk ratio with 95% confidence interval, information on heterogeneity, information on publication bias, and systematic review authors’ assessment and grading of the quality of evidence by using the NutriGrade [13] scoring system, if reported. One reviewer (M.U.J.) extracted the data and a second reviewer (H.M.) checked the extracted data. Disagreements were resolved by discussion. We did not contact review authors at all, even if data were missing from systematic reviews, inadequately reported in systematic reviews, or reported differently across systematic reviews.

### 2.5. Assessment of the Quality of How the Systematic Reviews were Conducted

The quality of how each systematic review was conducted was assessed and the overall confidence in the results of each systematic review was rated independently by two reviewers (M.U.J. and E.T.) using the AMSTAR 2 [12] appraisal tool, as detailed in Supplementary Materials Methods S2. Any disagreement between reviewers was resolved by discussion.

### 2.6. Evidence Synthesis from Meta-Analyses Contained within the Systematic Reviews

Evidence of risk of CVD and major subtypes of CVD associated with the intake of unprocessed and processed meat from primary study meta-analyses included in the systematic reviews was summarized. For linear dose-response meta-analysis, we converted effect estimates to express risk per 50 g/d higher intake, if reported differently. Effect estimates from low versus high intake meta-analysis were converted to express high versus low intake. The meta-evidence (defined as the quality of evidence of meta-analyses: confidence in the estimate) of primary studies was assessed and graded independently by two reviewers (M.U.J. and A.B.) using the NutriGrade [13] scoring system, as detailed in Supplementary Materials Methods S3. If a high versus low (or a low versus high) intake meta-analysis as well as a dose-response meta-analysis was available for an exposure, we assessed and graded the quality of evidence of the dose-response meta-analysis. Any disagreement between reviewers was resolved by discussion.

## 3. Results

### 3.1. Study Selection

**Figure 1:**
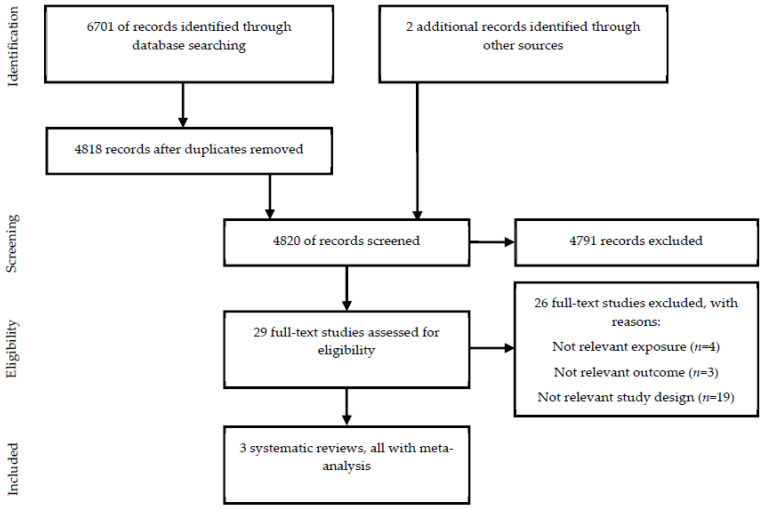
Flow diagram summarizing the study selection processes.

Out of the 6703 records identified through database searching and other sources, 29 full-text studies were assessed for eligibility and three systematic reviews [17,18,19] met our eligibility criteria (Figure 1). The entire list of the 26 full-text studies excluded [20,21,22,23,24,25,26,27,28,29,30,31,32,33,34,35,36,37,38,39,40,41,42,43,44,45], including reasons, can be found in Supplementary Materials Table S1.

### 3.2. Description of Included Reviews

We identified systematic reviews on the following exposures: intake of unprocessed red meat [17,19], intake of unprocessed poultry [17], and intake of processed meat [17,18,19]. Supplementary Materials Table S2 shows the definition of meat categories as described in the reviews. Descriptive characteristics of the included systematic reviews, sorted by the exposure, are shown in Table 1, Table 2 and Table 3. The systematic reviews were published between 2017 and 2019 and included meta-analyses of primary studies on the associations between unprocessed red meat and CVD (*n* = 1) or stroke (*n* = 2) (Table 1), unprocessed poultry and stroke (*n* = 1) (Table 2), and processed meat and CVD (*n* = 1), CHD (*n* = 1), or stroke (*n* = 3) (Table 3).

Descriptive characteristics of the primary studies contained within included systematic reviews, sorted by the exposure, can be found in Supplementary Materials Tables S3–S5. The primary studies were from Asia, Australia, Europe, and North America and most studies included both men and women. Tables mapping the primary studies contained within the included systematic reviews can be found in Supplementary Materials Tables S6–S11. Primary studies overlapped in systematic reviews summarizing the findings on associations between unprocessed red meat and stroke (*n* = 2) (Supplementary Materials Table S7) and in systematic reviews summarizing findings on associations between processed meat and stroke (*n* = 3) (Supplementary Materials Table S11).

### 3.3. Assessment of the Quality of How the Systematic Reviews were Conducted

Supplementary Materials Table S12 provides a breakdown of how each systematic review was assessed on the domain-specific questions of the AMSTAR 2 appraisal tool and the rationale behind the assessments of the seven domains that can critically affect the validity of a review and its conclusions. In each systematic review, two critical flaws were present. Critical flaws were lack of protocol registered before the commencement of the review (*n* = 1), lack of justification for excluding individual studies (*n* = 2), lack of satisfactory technique for assessment of the risk of bias in primary studies (*n* = 1), and lack of assessment of presence and likely impact of publication bias (*n* = 2). Table 1, Table 2 and Table 3 show the rating of the overall confidence in the results of each systematic review.

### 3.4. Evidence Synthesis from Meta-Analyses Contained within the Systematic Reviews

Supplementary Materials Tables S13–S15 provide the scoring for the different items of the NutriGrade scoring system for the primary study meta-analyses. Table 4, Table 5 and Table 6 show the summary risk ratio of each meta-analysis and the grading of the quality of evidence.

Moderate quality of evidence was found for a higher intake of unprocessed red meat, which was associated with a higher risk of stroke (Table 4). No clear association between intake of unprocessed red meat and risk of CVD was found from evidence graded as very low quality (Table 4).

A higher intake of unprocessed poultry associated with a lower risk of stroke was found from evidence graded as low quality (Table 5).

Moderate quality of evidence was observed for a higher intake of processed meat, which was associated with a higher risk of CHD and stroke (Table 6). No clear association between intake of processed meat and risk of CVD was found from evidence graded as very low quality (Table 6).

Summary risk ratios from high versus low intake meta-analyses were in the same direction as the summary risk ratios from the dose-response analyses (Supplementary Materials Tables S16–S18).

## 4. Discussion

In our overview, we summarized the current body of systematic review evidence on intake of unprocessed and processed meat and the risk of CVD and major subtypes of CVD. We identified three systematic reviews including evidence of primary study meta-analyses of associations with risk of CVD, CHD, and stroke for unprocessed red meat, unprocessed poultry, and processed meat intake. The systematic reviews were published between 2017 and 2019 and addressed related review questions. They included meta-analyses of primary studies on the associations between unprocessed red meat and CVD or stroke, unprocessed poultry and stroke, and processed meat and CVD, CHD, or stroke. We assessed the quality of how each review was conducted and the quality of evidence of each meta-analysis contained within the systematic reviews. Each review was rated as critically low, which means that ‘the systematic review has more than one critical flaw and should not be relied on to provide an accurate and comprehensive summary of the available studies’ [12]. Meta-evidence was graded as moderate for intake of unprocessed red meat, which was associated with higher risk of stroke, and moderate for intake of processed meat, which was associated with higher risk of CHD and stroke. This means that there is moderate confidence in the effect estimates; further research could add evidence on the confidence in the effect estimates and may change the effect estimates [13]. For the other associations, the quality of evidence was graded low or very low.

### 4.1. Strengths and Limitations

A major strength of our overview is the systematic quality assessment of the conduct of systematic reviews investigating intake of unprocessed and processed meat in relation to development of CVD and major subtypes of CVD. However, there are some inherited limitations when summarizing the current body of systematic review evidence on a topic. The conclusions rely on the quality of how the included systematic reviews were conducted and the methodological quality of the primary studies contained within the systematic reviews.

We used the well-established AMSTAR 2 [12] appraisal tool to enable critical appraisal of the conduct of included systematic reviews. We found it, however, relevant to deviate from the tool in addressing some of the domain-specific questions. We decided not to include ‘searched study registries’ in our assessment of the approach used to identify studies that meet the eligibility criteria for the systematic reviews. This was because most cohort studies (contemporary studies) are not registered before being conducted. Furthermore, ‘included/consulted content experts in the field’ was not considered relevant because only a biased sample of such studies can be identified and because unpublished studies may tend to be of lower quality. Also, we did not include ‘searched for grey literature’ as grey literature may not have been subject to peer review and therefore may be of lower quality. ‘Selection of the reported result from among multiple measurements or analyses of a specified outcome’ was not included in our assessment of the technique used for assessment of the risk of bias in primary studies contained within the systematic reviews. This was also because most cohort studies are not registered before being conducted.

In each of the three included reviews [17,18,19], two critical flaws were present. Kim et al. [17] did not state that they had a written protocol or guide that included the review question and methods. Adherence to a well-developed protocol reduces the risk of bias in the review [12]. Thus, the methods for the review should be agreed on before the review commence. Furthermore, the authors did not provide a list of all potentially relevant studies that were retrieved and assessed for eligibility but excluded from the review, with reasons for such. Excluded studies should be accounted for fully by review authors, otherwise there is a risk that the studies remain invisible and the impact of their exclusion from the review is unknown [12]. However, the authors provided a PRISMA flow diagram with information about the number of studies excluded after full-text screening, by reason. In line with Kim et al. [17], Zeraatkar et al. [19] provided a PRISMA flow diagram with information about the number of studies excluded after full-text screening, by reason, but not a complete list of potentially relevant studies that were excluded from the review, with justifications. Bechthold et al. [18] did not use a satisfactory technique for assessing the risk of bias in the primary studies that were included in the review. They used the risk of bias tool integrated within the NutriGrade [13] scoring system which includes items concerning information bias, confounding, and follow-up duration but not sample selection bias. However, the AMSTAR 2 [12] appraisal tool does not differentiate between different observational study designs, and because the primary studies were cohort studies, sample selection bias is not of major concern. Thus, it could be argued that the overall confidence in the results of the review by Bechthold et al. [18] should not be rated down due to lack of assessment of risk of bias from sample selection in primary studies. Bechthold et al. [18] and Zeraatkar et al. [19] did not address publication bias. Typically, funnel plots and statistical tests for examining funnel plot asymmetry are used to detect publication bias [46]. If the results of the statistical tests to detect publication bias are positive (small *p*-values), they indicate evidence for publication bias. On the other hand, negative test results (large *p*-values) do not indicate that publication bias can be safely ignored. Insignificance of tests used for examining funnel plot asymmetry should be trusted only when there are at least 10 studies included in the meta-analysis, because when there are fewer studies, the power of the tests is low [46]. The maximum number of primary studies in the meta-analyses contained within the included reviews [17,18,19] was six.

We extracted the risk of bias (methodological quality) assessment that was presented in each included systematic review and provided tabular summaries of the assessments. The included systematic reviews [17,18,19] used different tools to assess risk of bias in the primary studies. In the systematic review by Kim et al. [17], the Newcastle-Ottawa Scale (NOS) [47] tool was used to assess the methodological quality of the primary studies contained within the review. The NOS scores of the primary studies varied between seven and eight stars (out of nine possible). The NOS is a commonly used tool for methodological quality assessment of cohort studies [10], but the tool’s discriminatory ability is questionable, as also demonstrated by Bae [48]. Out of 89 cohort studies on dietary intake and incidence of various cancers included in 14 systematic reviews with quality assessment using the NOS tool, 81 studies (91%) were considered of high methodological quality (low risk of bias) [48]. Lack of variable quality assessment of the primary studies may explain why Kim et al. [17] did not assess the potential impact of risk of bias in primary studies on the results of their meta-analyses. But they discussed the likely impact of risk of bias on the results. In the two other systematic reviews [18,19], less common tools were used to assess the risk of bias in the primary studies. Bechthold et al. [18] used the risk of bias tool integrated within the NutriGrade [13] scoring system, and Zeraatkar et al. [19] used the Clinical Advances Through Research and Information Translation (CLARITY) [49] tool. The most comprehensive tool to assess the risk of bias in observational studies is the Risk of Bias in Non-randomized Studies—of Interventions (ROBINS-I) [50] tool [12], released in 2016, and a modified version to assess the risk of bias in non-randomized studies of exposures is under development [51]. Scientists have begun to validate ROBINS-I, and experience will accumulate [52].

We used the NutriGrade [13] scoring system to assess and grade the quality of evidence of the meta-analyses contained within the systematic reviews. This was because the NutriGrade scoring system was developed to evaluate the quality of cohort study meta-analyses in nutrition research [13]. However, a limitation of the scoring system is the circular reasoning. NutriGrade assumes that there is an effect on outcome of the exposure because the tool scores the demonstration of an effect. For example, item 8: ‘a dose-response association increases the confidence in the findings of cohort studies and thus enhances the assigned meta-evidence’ [13]. If there indeed is no causal effect of an exposure on the outcome, and no effect (association) is detected, a total score of 7 points out of a maximum of 10 points can at most be reached. Thus, the meta-analysis may have correctly uncovered a lack of effect, with maximum points in all other NutriGrade items than the items that scores the demonstration of an effect. Yet, the grading of the quality of the evidence is translated to ‘moderate’, solely because of the lack of an effect demonstration; an effect which may not be present. While the lack of a possibility to detect an effect due to statistical imprecision (item 2) is a natural point of criticism, the fact that an effect may be absent, and not just undetected, compromises the NutriGrade score as one of both quality and effect demonstration. In other words, if an effect is absent, it is not a quality of a meta-analysis to detect such. However, at present, we still consider the NutriGrade scoring system the best available tool to assess the quality of cohort study meta-analyses in nutrition research. The Grading of Recommendations Assessment, Development, and Evaluation (GRADE) working group [53] has developed a widely accepted system to rate the certainty of a body of evidence in the contexts of systematic reviews, developing health-care recommendations, and supporting decisions [52]. In 2018, the working group provided guidance on how systematic review authors should approach the use of the ROBINS-I [50] tool as part of GRADE’s certainty rating process [52]. Due to the integration of ROBINS-I within the GRADE system, experts within the field of public health nutrition have suggested the GRADE system to rate the certainty of the systematic review evidence of observational studies on diet and disease outcomes [10]. However, as stated above, a tool to assess the risk of bias in non-randomized exposure studies is under development.

### 4.2. Comparison with Existing Dietary Guidelines

Moderate quality of evidence was found for a higher intake of unprocessed red meat, which was associated with a higher risk of stroke, and for a higher intake of processed meat, which was associated with a higher risk of CHD and stroke. However, in the review by Kim et al. [17], the meta-evidence was judged ‘low’ for a positive association between unprocessed red meat and stroke and ‘low’ for a positive association between processed meat and stroke. This inconsistency in grading of quality of evidence was primarily due to differences in the type of comparisons in the meta-analyses. Bechthold et al. [18] and Zeraatkar et al. [19] conducted dose-response meta-analysis while Kim et al. [17] carried out high versus low intake meta-analysis. In all meta-analyses, the corresponding tests were statistically significant, but any type of dose-response association is an important factor for the presence of a causal effect [54], which is why the meta-evidence was graded ‘moderate’ in the reviews by Bechthold et al. [18] and Zeraatkar et al. [19] and ’low’ in the review by Kim et al. [17]. Thus, our overview indicates that the current body of systematic review evidence on intake of unprocessed and processed meat and the risk of CVD and major subtypes of CVD supports existing dietary guidelines; however, none of the reviews addressed associations with lean versus fat meat. For example, the Nordic Nutrition Recommendations 2012 [55] recommend to decrease consumption of red meat and processed meat, and the Dietary Guidelines for Americans 2020-2025 recommends that ‘most intake of meats should be from fresh, frozen, or canned, and in lean forms (e.g., chicken breast or ground turkey) versus processed meats (e.g., hot dogs, sausages, ham, luncheon meats)’ [56]. But each review was rated as critically low, which means that ‘the systematic review has more than one critical flaw and should not be relied on to provide an accurate and comprehensive summary of the available studies’ [12]. Furthermore, none of the reviews addressed findings from primary studies on specified food substitution analyses to identify optimal food composition of the diet; probably because of the lack of specified food substitution analyses in most cohort studies on diet-disease relationships [57]. Food substitution analysis, however, is highly relevant to inform public health policy decision-makers [57].

## 5. Conclusions and Future Research Directions

There was moderate quality of evidence of meta-analyses for unprocessed red meat, which was associated with a higher risk of stroke, and moderate quality of evidence of meta-analyses for processed meat, which was associated with a higher risk of CHD and stroke. But critical weaknesses were detected in the systematic reviews. Overall, the associations between unprocessed and processed meat with CVD and major subtypes of CVD have not been extensively investigated. Future studies should investigate substitutions between different meat subtypes and between meat subtypes and other sources of protein and micronutrients, of which meat is an important source, to identify optimal food composition of the diet. Specifically, studies on substitutions between meat and other protein sources may be relevant to inform public health policy decision-makers due to the lower climate impact of plant-based diets [58,59].

## Figures and Tables

**Table 1:** Descriptive characteristics of systematic reviews on associations between unprocessed red meat and CVD and stroke.

First Author’s Last Name and Publication Year	Outcome	Databases Searched	Date Ranges of Databases Searched	Meta- Analysis					Did the Review Authors Report any Potential Sources of Conflict of Interest, Including Any Funding They Received for Conducting the Review? ^1^	AMSTAR 2 Rating of Overall Confidence in the Results of the Systematic Review
				Type of Comparison	Number of Cohort Studies	Number of Participants	Number of Events	Authors’ Conclusion n		
Zeraatkar 2019 [19]	CVD	Medline; Embase; Cochrane Library; Web of Science; CINAHL; ProQuest	From inception until July 2018 (for MEDLINE from inception until April 2019)	Low versus high intake	4	65,736	n/a	n/a	The authors described their fun-ding sources and how they managed potential conflicts of interest	Critically low ^2^ (due to lack of justification for excluding individual studies and lack of assessment of presence and likely impact of publication bias)
				Dose-response (per lower intake)	3	191,803	52,765	No association		
Kim 2017 [17]	Stroke	PubMed; Embase; Cochrane Library	Through October 2016	High versus low intake	6	254,742	9522	Positive association	The authors reported no competing interests	Critically low ^2^ (due to lack of protocol registered before the commencement of the review and lack of justification for excluding individual studies)
Zeraatkar 2019 [19]	Stroke	Medline; Embase; Cochrane Library; Web of Science; CINAHL; ProQuest	From inception until July 2018 (for MEDLINE from inception until April 2019)	Low versus high intake	6	102,024	n/a	n/a	The authors described their fun-ding sources and how they managed potential conflicts of interest	Critically low ^2^ (due to lack of justification for excluding individual studies and lack of assessment of presence and likely impact of publication bias)
				Dose-response (per lower intake)	6	254,742	13,113	Negative association		

CVD, indicates cardiovascular disease; n/a, not provided, because the answer is not available from the systematic review. ^1^ Item 16 in AMSTAR 2. ^2^ The systematic review has more than one critical flaw and should not be relied on to provide an accurate and comprehensive summary of the available studies.

**Table 2:** Descriptive characteristics of systematic reviews on associations between unprocessed poultry and stroke.

First Author’s Last Name and Publication Year	Outcome	Databases Searched	Date Ranges of Databases Searched	Meta- Analysis					Did the Review Authors Report any Potential Sources of Conflict of Interest, Including Any Funding they Received for Conducting the Review? ^1^	AMSTAR 2 Rating of Overall Confidence in the Results of the Systematic Review
				Type of Comparison	Number of Cohort Studies	Number of Participants	Number of Events	Authors’ Conclusion		
Kim 2017 [17]	Stroke	PubMed; Embase; Cochrane Library	Through October 2016	High versus low intake	3	138,761	4759	Negative association	The authors reported no competing interests	Critically low ^2^ (due to lack of protocol registered before the commencement of the review and lack of justification for excluding individual studies)

^1^ Item 16 in AMSTAR 2. ^2^ The systematic review has more than one critical flaw and should not be relied on to provide an accurate and comprehensive summary of the available studies.

**Table 3:** Descriptive characteristics of systematic reviews on associations between processed meat and CVD, CHD, and stroke.

First Author’s Last Name and Publication Year	Outcome	Databases Searched	Date Ranges of Databases Searched	Meta- Analysis					Did the Review Authors Report any Potential Sources of Conflict of Interest, Including Any Funding They Received for Conducting the Review? ^1^	AMSTAR 2 Rating of Overall Confidence in the Results of the Systematic Review
				Type of Comparison	Number of Cohort Studies	Number of Participants	Number of Events	Authors’ Conclusion		
Zeraatkar 2019 [19]	CVD	Medline; Embase; Cochrane Library; Web of Science; CINAHL; ProQuest	From inception until July 2018 (for MEDLINE from inception until April 2019)	Low versus high intake	4	69,186	n/a	n/a	The authors describ-ed their funding sources and how they managed potential conflicts of interest	Critically low ^2^ (due to lack of justification for excluding individual studies and lack of assessment of presence and likely impact of publication bias)
				Dose-response (per lower intake)	3	200,421	52,765	No association		
Bechthold 2019 [18]	CHD	PubMed; Embase	Until March 2017	High versus low intake	5	196,820	7038	Positive association	The authors reported no competing interests	Critically low ^2^ (due to lack of satisfactory technique for assessment of the risk of bias in primary studies and lack of assessment of presence and likely impact of publication bias)
				Dose-response (per higher intake)	3	151,373	6659	Positive association		
Kim 2017 [17]	Stroke	PubMed; Embase; Cochrane Library	Through October 2016	High versus low intake	6	254,742	9522	Positive association	The authors reported no competing interests	Critically low ^2^ (due to lack of protocol registered before the commencement of the review and lack of justification for excluding individual studies)
Bechthold 2019 [18]	Stroke	PubMed; Embase	Until March 2017	High versus low intake	6	254,742	9492	Positive association	The authors reported no competing interests	Critically low ^2^ (due to lack of satisfactory technique for assessment of the risk of bias in primary studies and lack of assessment of presence and likely impact of publication bias)
				Dose-response (per higher intake)	6	254,742	9492	Positive association		
Zeraatkar 2019 [19]	Stroke	Medline; Embase; Cochrane Library; Web of Science; CINAHL; ProQuest	From inception until July 2018 (for MEDLINE from inception until April 2019)	Low versus high intake	6	101,861	n/a	n/a	The authors describ-ed their funding sources and howthey managed potential conflicts of interest	Critically low ^2^ (due to lack of justification for excluding individual studies and lack of assessment of presence and likely impact of publication bias)
				Dose-response (per lower intake)	6	254,742	13,113	Negative association		

CHD, indicates coronary heart disease; CVD, cardiovascular disease; n/a, not provided, because the answer is not available from the systematic review. ^1^ Item 16 in AMSTAR 2. ^2^ The systematic review has more than one critical flaw and should not be relied on to provide an accurate and comprehensive summary of the available studies.

**Table 4:** Meta-analyses of associations between intake of unprocessed red meat and risk of CVD and stroke, and NutriGrade meta-evidence grading.

First Author’s Last Name and Publication Year	Out-come	Number of Cohort Studies	Type of Comparison	Summary Risk Ratio (95% CI)	Heterogeneity (I^2^)	NutriGrade Score ^1^	NutriGrade Grading of Quality of Evidence ^1^
Zeraatkar 2019 [19]	CVD	3	Dose-response (per 50 g/day higher intake)	1.05 (0.94, 1.17) ^2^	37.2%	3.0	Very low ^3^
Kim 2017 [17]	Stroke	6	High versus low intake	1.11 (1.03, 1.20)	0.0%	5.9	Low ^4^
Zeraatkar 2019 [19]	Stroke	6	Dose-response (per 50 g/day higher intake)	1.06 (1.02, 1.11) ^2^	0.0%	6.4	Moderate ^5^

CI, indicates confidence interval; CVD, cardiovascular disease. ^1^ The scoring system is numerical (maximum of 10 points) and includes eight items: Risk of bias, study quality, and study limitation (item 1, maximum of 2 points), Precision (item 2, maximum of 1 point), Heterogeneity (item 3, maximum of 1 point), Directness of evidence (item 4, maximum of 1 point), Publication bias (item 5, maximum of 1 point), Funding bias (item 6, maximum of 1 point), Effect size (item 7, maximum of 2 points), and Dose-response (item 8, maximum of 1 point). Four categories are established to grade the quality of evidence of meta-analyses. A score of ≥8 points is assigned to high, 6 to <8 points to moderate, 4 to <6 points to low, and <4 points to very low. In Zeraatkar 2019, scoring for effects size was based on summary risk ratio estimates from dose-response meta-analysis. ^2^ Risk ratio converted from 3 servings/week lower intake to 50 g/day higher intake by using the formula $RR_{+50g/day}=\frac{1}{\left[{\left(RR_{-3x120g/week}\right)}^{\frac{7x50}{3x120}}\right]}$ assuming that each serving of unprocessed red meat was equal to 120 g; the same amount as used by Zeraatkar 2019 for primary studies reporting exposure as number of servings. ^3^ There is very low confidence in the effect estimate; meta-evidence is very limited and uncertain. ^4^ There is low confidence in the effect estimate; further research will provide important evidence on the confidence and likely change the effect estimate. ^5^ There is moderate confidence in the effect estimate; further research could add evidence on the confidence and may change the effect estimate.

**Table 5:** Meta-analyses of associations between intake of unprocessed poultry and risk of stroke, and NutriGrade meta-evidence grading.

First Author’s Last Name and Publication Year	Out-come	Number of Cohort Studies	Type of Comparison	Summary Risk Ratio (95% CI)	Heterogeneity (I^2^)	NutriGrade Score ^1^	NutriGrade Grading of Quality of Evidence ^1^
Kim 2017 [17]	Stroke	3	High versus low intake	0.87 (0.78, 0.96)	0.0%	5.0	Low ^2^

CI, indicates confidence interval. ^1^ The scoring system is numerical (maximum of 10 points) and includes eight items: Risk of bias, study quality, and study limitation (item 1, maximum of 2 points), Precision (item 2, maximum of 1 point), Heterogeneity (item 3, maximum of 1 point), Directness of evidence (item 4, maximum of 1 point), Publication bias (item 5, maximum of 1 point), Funding bias (item 6, maximum of 1 point), Effect size (item 7, maximum of 2 points), and Dose-response (item 8, maximum of 1 point). Four categories are established to grade the quality of evidence of meta-analyses. A score of ≥8 points is assigned to high, 6 to <8 points to moderate, 4 to <6 points to low, and <4 points to very low. ^2^ There is low confidence in the effect estimate; further research will provide important evidence on the confidence and likely change the effect estimate.

**Table 6:** Meta-analyses of associations between intake of processed meat and risk of CVD, CHD, and stroke, and NutriGrade meta-evidence grading.

First Author’s Last Name and Publication Year	Out-come	Number of Cohort Studies	Type of Comparison	Summary risk ratio (95% CI)	Heterogeneity (I^2^)	NutriGrade Score ^1^	NutriGrade Grading of Quality of Evidence ^1^
Zeraatkar 2019 [19]	CVD	3	Dose-response (per 50 g/day higher intake)	1.07 (0.82, 1.38) ^2^	59.6%	3.0	Very low ^3^
Bechthold 2019 [18]	CHD	3	Dose-response (per 50 g/day higher intake)	1.27 (1.09, 1.49)	0.0%	n/a	Moderate ^4,5^
Kim 2017 [17]	Stroke	6	High versus low intake	1.17 (1.08, 1.25)	0.0%	5.9	Low ^6^
Bechthold 2019 [18]	Stroke	6	Dose-response (per 50 g/day higher intake)	1.17 (1.02, 1.34)	56%	n/a	Moderate ^4,5^
Zeraatkar 2019 [19]	Stroke	6	Dose-response (per 50 g/day higher intake)	1.16 (1.05, 1.28) ^2^	40.2%	6.2	Moderate ^4^

CHD, indicates coronary heart disease; CI, confidence interval; CVD, cardiovascular disease; n/a, not provided, because the answer is not available from the systematic review. ^1^ The scoring system is numerical (maximum of 10 points) and includes eight items: Risk of bias, study quality, and study limitation (item 1, maximum of 2 points), Precision (item 2, maximum of 1 point), Heterogeneity (item 3, maximum of 1 point), Directness of evidence (item 4, maximum of 1 point), Publication bias (item 5, maximum of 1 point), Funding bias (item 6, maximum of 1 point), Effect size (item 7, maximum of 2 points), and Dose-response (item 8, maximum of 1 point). Four categories are established to grade the quality of evidence of meta-analyses. A score of ≥8 points is assigned to high, 6 to <8 points to moderate, 4 to <6 points to low, and <4 points to very low. In Zeraatkar 2019, scoring for effects size was based on summary risk ratio estimates from dose-response meta-analysis. ^2^ Risk ratio converted from 3 servings/week lower intake to 50 g/day higher intake by using the formula $RR_{+50g/day}=\frac{1}{\left[{\left(RR_{-3x50g/week}\right)}^{\frac{7x50}{3x50}}\right]}$ assuming that each serving of processed meat was equal to 50 g; the same amount as used by Zeraatkar 2019 for primary studies reporting exposure as number of servings. ^3^ There is very low confidence in the effect estimate; meta-evidence is very limited and uncertain. ^4^ There is moderate confidence in the effect estimate; further research could add evidence on the confidence and may change the effect estimate. ^5^ Systematic review authors’ grading. ^6^ There is low confidence in the effect estimate; further research will provide important evidence on the confidence and likely change the effect estimate.

## Supplementary Materials

The following are available online at https://www.mdpi.com/article/10.3390/nu13103303/s1, Methods S1: Literature search, Methods S2: Assessment of the quality of how the systematic reviews were conducted, Methods S3: Assessment of the quality of evidence of meta-analyses contained within the included systematic reviews, Table S1: Full-text studies excluded, Table S2: Definition of meat as described in systematic reviews on associations between unprocessed and processed meat and CVD and major subtypes of CVD, Table S3: Descriptive characteristics of primary studies contained within systematic reviews on intake of unprocessed red meat and risk of CVD and stroke, Table S4: Descriptive characteristics of primary studies contained within systematic reviews on intake of unprocessed poultry and risk of stroke, Table S5: Descriptive characteristics of primary studies contained within systematic reviews on intake of processed meat and risk of CVD, CHD, and stroke, Table S6: Primary studies contained within systematic reviews on intake of unprocessed red meat and risk of CVD, Table S7: Primary studies contained within systematic reviews on intake of unprocessed red meat and risk of stroke, Table S8: Primary studies contained within systematic reviews on intake of unprocessed poultry and risk of stroke, Table S9: Primary studies contained within systematic reviews on intake of processed meat and risk of CVD, Table S10: Primary studies contained within systematic reviews on intake of processed meat and risk of CHD, Table S11: Primary studies contained within systematic reviews on intake of processed meat and risk of stroke, Table S12: Assessment for the different items of AMSTAR 2 and rationale behind assessment for the critical domains of AMSTAR 2, Table S13: Scoring for the different items of NutriGrade (maximum of 10 points) for primary study meta-analyses of unprocessed red meat, Table S14: Scoring for the different items of NutriGrade (maximum of 10 points) for primary study meta-analyses of unprocessed poultry, Table S15: Scoring for the different items of NutriGrade (maximum of 10 points) for primary study meta-analyses of processed meat, Table S16: High versus low meta-analyses of associations between intake of unprocessed red meat and risk of CVD and stroke, Table S17: High versus low meta-analyses of associations between intake of unprocessed poultry and risk of stroke, Table S18: High versus low meta-analyses of associations between intake of processed meat and risk of CVD, CHD, and stroke.
