# Supplementary material for: Intake of Unprocessed and Processed Meat and the Association with Cardiovascular Disease: An Overview of Systematic Reviews
Source: Nutrients. 2021 Sep 22;13(10):3303. doi: 10.3390/nu13103303 (PMC8539526; doi:10.3390/nu13103303)
Supplement: Supplementary file 1 [file nutrients-13-03303-s001.zip › nutrients-1385389-supplementary.pdf]

Supplementary Materials

Intake of unprocessed and processed meat and the association with cardiovascular disease: An overview of systematic reviews

Marianne Uhre Jakobsen*, Anette Bysted, Heddie Mejborn, Anders Stockmarr, Ellen Trolle

*Correspondence to muja@food.dtu.dk

Methods S1. Literature search

Studies were identified through a systematic literature search in the bibliographic databases PubMed, Embase, and Web of Science. The search included only terms related to exposures and outcomes.

Search strategy for PubMed:

Exposures:

#1: meat[MeSH Terms] OR meat[Title/Abstract]

Outcomes:

#2: cardiovascular diseases[MeSH Terms] OR cardiovascular[Title/Abstract] OR CVD[Title/Abstract] OR ischemic heart[Title/Abstract] OR acute coronary syndrome[Title/Abstract] OR coronary artery[Title/Abstract] OR coronary heart[Title/Abstract] OR CHD[Title/Abstract] OR myocardial infarction[Title/Abstract] OR sudden cardiac[Title/Abstract] OR stroke[Title/Abstract]

Exposures and outcomes combined:

#3: #1 AND #2

Exposures, outcomes, and data range combined:

#4: #3 AND '2010/08/25'[Date - Publication] : '3000'[Date - Publication]

The search terms were adapted for use with Embase and Web of Science.

Methods S2. Assessment of the quality of how the systematic reviews were conducted

The AMSTAR 2 [1] appraisal tool was used to assess the quality of how each included systematic review was conducted and to rate the overall confidence in the results of each systematic review. The tool includes 16 items about how the systematic review was conducted. The domain-specific questions can be answered with 'Yes' (for items 2, 4, and 7–9 'Yes' or 'Partial Yes') or 'No' (for items 11, 12, and 15 'No' or 'No meta-analysis conducted') and are framed so that a 'Yes' answer denotes a positive result. All steps in the conduct of a systematic review and meta-analysis are important, but seven domains can critically affect the validity of a review and its conclusions [1]. These seven critical domains are: Protocol registered before the commencement of the review (item 2), Adequacy of the literature search (item 4), Justification for excluding individual studies (item 7), Risk of bias from primary studies being included in the review (item 9), Appropriateness of meta-analytical methods (item 11), Consideration of risk of bias when interpreting the results of the review (item 13), and Assessment of presence and likely impact of publication bias (item 15). If a high versus low (or a low versus high) intake meta-analysis as well as a dose-response meta-analysis was available for an exposure, we assessed the dose-response meta-analysis.

Four categories are recommended to rate the overall confidence in the results. If no or one non-critical weakness is present, the overall confidence in the results is *high*, which means that 'the systematic review provides an accurate and comprehensive summary of the results of the available studies that address the question of interest' [1]. If more than one non-critical weakness is present, the overall confidence in the results is *moderate*, which means that 'the systematic review has more than one weakness but no critical flaws. It may provide an accurate summary of the results of the available studies that were included in the review' [1]. If one critical flaw with or without non-critical weaknesses is present, the overall confidence in the results is *low*, which means that 'the review has a critical flaw and may not provide an accurate and comprehensive summary of the available studies that address the question of interest' [1]. In case of more than one critical flaw with or without non-critical weaknesses is present, the overall confidence in the results is *critically low*, which means that 'the review has more than one critical flaw and should not be relied on to provide an accurate and comprehensive summary of the available studies' [1].

References

1. Shea, B.J.; Reeves, B.C.; Wells, G.; Thuku, M.; Hamel, C.; Moran, J.; Moher, D.; Tugwell, P.; Welch, V.; Kristjansson, E.; et al. AMSTAR 2: A Critical Appraisal Tool for Systematic Reviews That Include Randomised or Non-Randomised Studies of Healthcare Interventions, or Both. *BMJ* **2017**, *358*, j4008, doi:10.1136/bmj.j4008.

Methods S3. Assessment of the quality of evidence of meta-analyses contained within the included systematic reviews

The NutriGrade [1] scoring system was used to assess and grade the meta-evidence (defined as the quality of evidence of meta-analyses: confidence in the estimate) of primary studies. The scoring system is numerical (maximum of 10 points) and includes eight items: Risk of bias, study quality, and study limitation (item 1, maximum of 2 points), Precision (item 2, maximum of 1 point), Heterogeneity (item 3, maximum of 1 point), Directness of evidence (item 4, maximum of 1 point), Publication bias (item 5, maximum of 1 point), Funding bias (item 6, maximum of 1 point), Effect size (item 7, maximum of 2 points), and Dose-response (item 8, maximum of 1 point).

Four categories are established to grade the quality of evidence of meta-analyses. If there is a score of ≥8 points, the quality of evidence is *high*, which means that 'there is high confidence in the effect estimate, and further research probably will not change the confidence in the effect estimate' [1]. If the score is 6 to <8 points, the quality of evidence is *moderate*, which means that 'there is moderate confidence in the effect estimate; further research could add evidence on the confidence and may change the effect estimate' [1]. If the score is 4 to <6 points, the quality of evidence is *low*, which means that 'there is low confidence in the effect estimate; further research will provide important evidence on the confidence and likely change the effect estimate' [1]. In case of a score of <4 points, the quality of evidence is *very low*, which means that 'there is very low confidence in the effect estimate; meta-evidence is very limited and uncertain' [1].

References

1. Schwingshackl, L.; Knüppel, S.; Schwedhelm, C.; Hoffmann, G.; Missbach, B.; Stelmach-Mardas, M.; Dietrich, S.; Eichelmann, F.; Kontopanteils, E.; Iqbal, K.; et al. Perspective: NutriGrade: A Scoring System to Assess and Judge the Meta-Evidence of Randomized Controlled Trials and Cohort Studies in Nutrition Research. *Adv. Nutr.* **2016**, *7*, 994–1004, doi:10.3945/an.116.013052.

Table S1. Full-text studies excluded

First author's last name and publication year	Reason			
	Population	Exposure	Outcome	Study design
McAfee 2010 [1]				1
Micha 2010 [2]				1
Bernstein 2011 [3]				1
Kaluza 2012 [4]			1	
Micha 2012 [5]				1
Åkesson 2013 [6]		1		
Chen 2013 [7]			1	
Feskens 2013 [8]				1
Foroughi 2013 [9]				1
Misirli 2015 [10]				1
Lippi 2015 [11]				1
Richi 2015 [12]				1
Boada 2016 [13]				1
Kouvari 2016 [14]				1
López-Romero 2016 [15]				1
Rohrmann 2016 [16]				1
Yang 2016 [17]			1	
Bronzato 2017 [18]				1
Larsson 2017 [19]				1
Micha 2017 [20]		1		
Wolk 2017 [21]				1
Ekmekcioglu 2018 [22]				1
Mohammadi 2018 [23]		1		
Lecerf 2019 [24]				1
Vernooij 2019 [25]		1		
Migliaccio 2020 [26]				1

References

1. McAfee, A.J.; McSorley, E.M.; Cuskelly, G.J.; Moss, B.W.; Wallace, J.M.W.; Bonham, M.P.; Fearon, A.M. Red Meat Consumption: An Overview of the Risks and Benefits. *Meat Sci.* **2010**, *84*, 1–13, doi:10.1016/j.meatsci.2009.08.029.
2. Micha, R.; Wallace, S.K.; Mozaffarian, D. Red and Processed Meat Consumption and Risk of Incident Coronary Heart Disease, Stroke, and Diabetes Mellitus. *Circulation* **2010**, *121*, 2271–2283, doi:10.1161/CIRCULATIONAHA.109.924977.
3. Bernstein, A.M.; Willett, W.C. Red Meat Intake and the Risk of Cardiovascular Disease. *Curr. Cardiovasc. Risk Rep.* **2011**, *5*, 145–148, doi:10.1007/s12170-010-0149-x.

4. Kaluza, J.; Wolk, A.; Larsson, S.C. Red Meat Consumption and Risk of Stroke. *Stroke* **2012**, *43*, 2556–2560, doi:10.1161/STROKEAHA.112.663286.

5. Micha, R.; Michas, G.; Mozaffarian, D. Unprocessed Red and Processed Meats and Risk of Coronary Artery Disease and Type 2 Diabetes – an Updated Review of the Evidence. *Curr. Atheroscler. Rep.* **2012**, *14*, 515–524, doi:10.1007/s11883-012-0282-8.

6. Åkesson, A.; Andersen, L.F.; Kristjánsdóttir, Á.G.; Roos, E.; Trolle, E.; Voutilainen, E.; Wirfält, E. Health Effects Associated with Foods Characteristic of the Nordic Diet: A Systematic Literature Review. *Food Nutr. Res.* **2013**, *57*, 22790, doi:10.3402/fnr.v57i0.22790.

7. Chen, G.-C.; Lv, D.-B.; Pang, Z.; Liu, Q.-F. Red and Processed Meat Consumption and Risk of Stroke: A Meta-Analysis of Prospective Cohort Studies. *Eur. J. Clin. Nutr.* **2013**, *67*, 91–95, doi:10.1038/ejcn.2012.180.

8. Feskens, E.J.M.; Sluik, D.; van Woudenbergh, G.J. Meat Consumption, Diabetes, and Its Complications. *Curr. Diab. Rep.* **2013**, *13*, 298–306, doi:10.1007/s11892-013-0365-0.

9. Foroughi, M.; Akhavanzanjani, M.; Maghsoudi, Z.; Ghiasvand, R.; Khorvash, F.; Askari, G. Stroke and Nutrition: A Review of Studies. *Int. J. Prev. Med.* **2013**, *4*, S165–S179.

10. Misirli, G.; Benetou, V.; Lagiou, P. [Nutrition as a Risk Factor for Cerebrovascular Disease]. *Arch. Hell. Med.* **2015**, *32*, 16–27.

11. Lippi, G.; Mattiuzzi, C.; Sanchis-Gomar, F. Red Meat Consumption and Ischemic Heart Disease. A Systematic Literature Review. *Meat Sci.* **2015**, *108*, 32–36, doi:10.1016/j.meatsci.2015.05.019.

12. Richi, E.B.; Baumer, B.; Conrad, B.; Darioli, R.; Schmid, A.; Keller, U. Health Risks Associated with Meat Consumption: A Review of Epidemiological Studies. *Int. J. Vitam. Nutr. Res.* **2015**, *85*, 70–78, doi:10.1024/0300-9831/a000224.

13. Boada, L.D.; Henríquez-Hernández, L.A.; Luzardo, O.P. The Impact of Red and Processed Meat Consumption on Cancer and Other Health Outcomes: Epidemiological Evidences. *Food Chem. Toxicol.* **2016**, *92*, 236–244, doi:10.1016/j.fct.2016.04.008.

14. Kouvari, M.; Tyrovolas, S.; Panagiotakos, D.B. Red Meat Consumption and Healthy Ageing: A Review. *Maturitas* **2016**, *84*, 17–24, doi:10.1016/j.maturitas.2015.11.006.

15. López-Romero, L.; Silva-Sieger, F.; Gamboa-Delgado, E. [Dietary Factors Associated with Stroke: A Literature Review]. *Rev. Neurol.* **2016**, *63*, 211–218.

16. Rohrmann, S.; Linseisen, J. Processed Meat: The Real Villain? *Proc. Nutr. Soc.* **2016**, *75*, 233–241, doi:10.1017/S0029665115004255.

17. Yang, C.; Pan, L.; Sun, C.; Xi, Y.; Wang, L.; Li, D. Red Meat Consumption and the Risk of Stroke: A Dose-Response Meta-Analysis of Prospective Cohort Studies. *J. Stroke Cerebrovasc. Dis.* **2016**, *25*, 1177–1186,

doi:10.1016/j.jstrokecerebrovasdis.2016.01.040.

18. Bronzato, S.; Durante, A. A Contemporary Review of the Relationship between Red Meat Consumption and Cardiovascular Risk. *Int. J. Prev. Med.* **2017**, *8*, 40, doi:10.4103/ijpvm.IJPVM_206_16.

19. Larsson, S.C. Dietary Approaches for Stroke Prevention. *Stroke* **2017**, *48*, 2905–2911, doi:10.1161/STROKEAHA.117.017383.

20. Micha, R.; Shulkin, M.L.; Peñalvo, J.L.; Khatibzadeh, S.; Singh, G.M.; Rao, M.; Fahimi, S.; Powles, J.; Mozaffarian, D. Etiologic Effects and Optimal Intakes of Foods and Nutrients for Risk of Cardiovascular Diseases and Diabetes: Systematic Reviews and Meta-Analyses from the Nutrition and Chronic Diseases Expert Group (NutriCoDE). *PLoS One* **2017**, *12*, e0175149, doi:10.1371/journal.pone.0175149.

21. Wolk, A. Potential Health Hazards of Eating Red Meat. *J. Intern. Med.* **2017**, *281*, 106–122, doi:10.1111/joim.12543.

22. Ekmekcioglu, C.; Wallner, P.; Kundi, M.; Weisz, U.; Haas, W.; Hutter, H.-P. Red Meat, Diseases, and Healthy Alternatives: A Critical Review. *Crit. Rev. Food Sci. Nutr.* **2018**, *58*, 247–261, doi:10.1080/10408398.2016.1158148.

23. Mohammadi, H.; Jayedi, A.; Ghaedi, E.; Golbidi, D.; Shab-Bidar, S. Dietary Poultry Intake and the Risk of Stroke: A Dose-Response Meta-Analysis of Prospective Cohort Studies. *Clin. Nutr. ESPEN* **2018**, *23*, 25–33, doi:10.1016/j.clnesp.2017.11.001.

24. Lecerf, J.-M. [Processed Meat and Cardio Metabolic Diseases]. *Cah. Nutr. Diet.* **2019**, *54*, S34–S40.

25. Vernooij, R.W.M.; Zeraatkar, D.; Han, M.A.; El Dib, R.; Zworth, M.; Milio, K.; Sit, D.; Lee, Y.; Gomaa, H.; Valli, C.; et al. Patterns of Red and Processed Meat Consumption and Risk for Cardiometabolic and Cancer Outcomes. *Ann. Intern. Med.* **2019**, *171*, 732–741, doi:10.7326/M19-1583.

26. Migliaccio, S.; Brasacchio, C.; Pivari, F.; Salzano, C.; Barrea, L.; Muscogiuri, G.; Savastano, S.; Colao, A. What Is the Best Diet for Cardiovascular Wellness? A Comparison of Different Nutritional Models. *Int. J. Obes. Suppl.* **2020**, *10*, 50–61, doi:10.1038/s41367-020-0018-0.

Table S2. Definition of meat as described in systematic reviews on associations between unprocessed and processed meat and CVD and major subtypes of CVD

First author's last name and publication year	Exposure	Exposure definition	Exposure in evidence synthesis
Kim 2017	Red meat	Unprocessed or fresh red meat	Unprocessed red meat
	White meat	Poultry meat only (fish excluded)	Unprocessed poultry[a]
	Processed meat	Processed meat or processed red meat	Processed meat
Bechthold 2019	Processed meat	n/a	Processed meat
Zeraatkar 2019	Unprocessed red meat	Mammalian meat	Unprocessed red meat
	Processed meat	White or red meat preserved by smoking, curing, salting, or adding chemical compounds (for example, hot dogs, charcuterie, sausage, ham, and deli meats)	Processed meat

CVD, indicates cardiovascular disease; n/a, not provided, because the answer is not available from the systematic review.
[a]Due to the authors' definition of processed meat, we derive that white meat includes only unprocessed white meat.

Table S3. Descriptive characteristics of primary studies contained within systematic reviews on intake of unprocessed red meat and risk of CVD and stroke

Systematic review		Descriptive characteristics				
First author's last name and publication year	Outcome	First author's last name and publication year	Cohort name	Study origin	Gender	Risk of bias assessment
Zeraatkar 2019	CVD	Bernstein 2010	NHS	US	Women	High risk
		von Ruesten 2013	EPIC-Potsdam	Germany	Combined	High risk
		Haring 2014	ARIC	US	Combined	Low risk
		Park 2017	KoGES	South Korea	Combined	High risk
Kim 2017	Stroke	Larsson 2011	Cohort of Swedish Men	Sweden	Men	NOS score 8
		Larsson 2011	Swedish Mammography Cohort	Sweden	Women	NOS score 8
		Bernstein 2012	HPFS	US	Men	NOS score 7
		Bernstein 2012	NHS	US	Women	NOS score 7
		Haring 2015	ARIC	US	Combined	NOS score 7
		Amiano 2016	EPIC-Spain	Spain	Combined	NOS score 7
Zeraatkar 2019	Stroke	Larsson 2011	Cohort of Swedish Men	Sweden	Men	Low risk
		Larsson 2011	Swedish Mammography Cohort	Sweden	Women	Low risk
		Bernstein 2012	HPFS	US	Men	Low risk
		Bernstein 2012	NHS	US	Women	Low risk
		Haring 2015	ARIC	US	Combined	High risk
		Amiano 2016	EPIC-Spain	Spain	Combined	High risk

ARIC, indicates Atherosclerosis Risk in Communities Study; CVD, cardiovascular disease; EPIC-Potsdam, European Prospective Investigation into Cancer and Nutrition-Potsdam; EPIC-Spain, European Prospective Investigation into Cancer and Nutrition-Spain; HPFS, Health Professionals Follow-Up Study; NHS, Nurses' Health Study; KoGES, Korean Genome and Epidemiology Study; NOS, Newcastle-Ottawa Scale.

Table S4. Descriptive characteristics of primary studies contained within systematic reviews on intake of unprocessed poultry and risk of stroke

Systematic review		Descriptive characteristics				
First author's last name and publication year	Outcome	First author's last name and publication year	Cohort name	Study origin	Gender	Risk of bias assessment
Kim 2017	Stroke	Bernstein 2012	HPFS	US	Men	NOS score 7
		Bernstein 2012	NHS	US	Women	NOS score 7
		Haring 2015	ARIC	US	Combined	NOS score 7

ARIC, indicates Atherosclerosis Risk in Communities Study; HPFS, Health Professionals Follow-Up Study; NHS, Nurses' Health Study; NOS, Newcastle-Ottawa Scale.

Table S5. Descriptive characteristics of primary studies contained within systematic reviews on intake of processed meat and risk of CVD, CHD, and stroke

Systematic review		Descriptive characteristics				
First author's last name and publication year	Outcome	First author's last name and publication year	Cohort name	Study origin	Gender	Risk of bias assessment
Zeraatkar 2019	CVD	Bernstein 2010	NHS	US	Women	High risk
		von Ruesten 2013	EPIC-Potsdam	Germany	Combined	High risk
		Haring 2014	ARIC	US	Combined	Low risk
		Son 2018	KoGES	South Korea	Combined	High risk
Bechthold 2019[a]	CHD	Ascherio 1994	HPFS	US	Men	Not reported
		Burke 2007	n/a	Australia	Combined	Not reported
		Bernstein 2010	NHS	US	Women	Not reported
		Haring 2014	ARIC	US	Combined	Not reported
		Würtz 2016	Diet, Cancer and Health	Denmark	Combined	Not reported
Kim 2017	Stroke	Larsson 2011	Cohort of Swedish Men	Sweden	Men	NOS score 8
		Larsson 2011	Swedish Mammography Cohort	Sweden	Women	NOS score 8
		Bernstein 2012	HPFS	US	Men	NOS score 7
		Bernstein 2012	NHS	US	Women	NOS score 7
		Haring 2015	ARIC	US	Combined	NOS score 7
		Amiano 2016	EPIC-Spain	Spain	Combined	NOS score 7
Bechthold 2019[a]	Stroke	Larsson 2011	Cohort of Swedish Men	Sweden	Men	Not reported
		Larsson 2011	Swedish Mammography Cohort	Sweden	Women	Not reported
		Bernstein 2012	HPFS	US	Men	Not reported
		Bernstein 2012	NHS	US	Women	Not reported
		Haring 2015	ARIC	US	Combined	Not reported
		Amiano 2016	EPIC-Spain	Spain	Combined	Not reported
Zeraatkar 2019	Stroke	Larsson 2011	Cohort of Swedish Men	Sweden	Men	Low risk
		Larsson 2011	Swedish Mammography Cohort	Sweden	Women	Low risk
		Bernstein 2012	HPFS	US	Men	Low risk
		Bernstein 2012	NHS	US	Women	Low risk
		Haring 2015	ARIC	US	Combined	High risk
		Amiano 2016	EPIC-Spain	Spain	Combined	High risk

ARIC, indicates Atherosclerosis Risk in Communities Study; CHD, coronary heart disease; CVD, cardiovascular disease; EPIC-Potsdam, European Prospective Investigation into Cancer and Nutrition-Potsdam; EPIC-Spain, European Prospective Investigation into Cancer and Nutrition-Spain; HPFS, Health Professionals Follow-Up Study; NHS, Nurses' Health Study; KoGES, Korean Genome and Epidemiology Study; NOS, Newcastle-Ottawa Scale.

[a]Risk of bias assessment of primary studies was conducted but not reported.

Table S6. Primary studies contained within systematic reviews on intake of unprocessed red meat and risk of CVD[a]

Primary study		Systematic review
First author's last name and publication year	Cohort name	Zeraatkar 2019
Bernstein 2010	NHS	X
von Ruesten 2013	EPIC-Potsdam	X
Haring 2014	ARIC	X
Park 2017	KoGES	X

ARIC, indicates Atherosclerosis Risk in Communities Study; CVD, cardiovascular disease; EPIC-Potsdam, European Prospective Investigation into Cancer and Nutrition-Potsdam; NHS, Nurses' Health Study; KoGES, Korean Genome and Epidemiology Study.

[a]X, indicates primary study included in systematic review.

Table S7. Primary studies contained within systematic reviews on intake of unprocessed red meat and risk of stroke[a]

Primary study		Systematic review	
First author's last name and publication year	Cohort name	Kim 2017	Zeraatkar 2019
Larsson 2011	Cohort of Swedish Men	X	X
Larsson 2011	Swedish Mammography Cohort	X	X
Bernstein 2012	HPFS	X	X
Bernstein 2012	NHS	X	X
Haring 2015	ARIC	X	X
Amiano 2016	EPIC-Spain	X	X

ARIC, indicates Atherosclerosis Risk in Communities Study; EPIC-Spain, European Prospective Investigation into Cancer and Nutrition-Spain; HPFS, Health Professionals Follow-Up Study; NHS, Nurses' Health Study.
[a]X, indicates primary study included in systematic review.

Table S8. Primary studies contained within systematic reviews on intake of unprocessed poultry and risk of stroke[a]

Primary study		Systematic review
First author's last name and publication year	Cohort name	Kim 2017
Bernstein 2012	HPFS	X
Bernstein 2012	NHS	X
Haring 2015	ARIC	X

ARIC, indicates Atherosclerosis Risk in Communities Study; HPFS, Health Professionals Follow-Up Study; NHS, Nurses' Health Study.

[a]X, indicates primary study included in systematic review.

Table S9. Primary studies contained within systematic reviews on intake of processed meat and risk of CVD[a]

Primary study		Systematic review
First author's last name and publication year	Cohort name	Zeraatkar 2019
Bernstein 2010	NHS	X
von Ruesten 2013	EPIC-Potsdam	X
Haring 2014	ARIC	X
Son 2018	KoGES	X

ARIC, indicates Atherosclerosis Risk in Communities Study; CVD, cardiovascular disease; EPIC-Potsdam, European Prospective Investigation into Cancer and Nutrition-Potsdam; NHS, Nurses' Health Study; KoGES, Korean Genome and Epidemiology Study.

[a]X, indicates primary study included in systematic review.

Table S10. Primary studies contained within systematic reviews on intake of processed meat and risk of CHD[a]

Primary study		Systematic review
First author's last name and publication year	Cohort name	Bechthold 2019
Ascherio 1994	HPFS	X
Burke 2007	n/a	X
Bernstein 2010	NHS	X
Haring 2014	ARIC	X
Würtz 2016	Diet, Cancer and Health	X

ARIC, indicates Atherosclerosis Risk in Communities Study; CHD, coronary heart disease; HPFS, Health Professionals Follow-Up Study; n/a, not provided, because the answer is not available from the systematic review; NHS, Nurses' Health Study.

[a]X, indicates primary study included in systematic review.

Table S11. Primary studies contained within systematic reviews on intake of processed meat and risk of stroke[a]

Primary study		Systematic review		
First author's last name and publication year	Cohort name	Kim 2017	Bechthold 2019	Zeraatkar 2019
Larsson 2011	Cohort of Swedish Men	X	X	X
Larsson 2011	Swedish Mammography Cohort	X	X	X
Bernstein 2012	HPFS	X	X	X
Bernstein 2012	NHS	X	X	X
Haring 2015	ARIC	X	X	X
Amiano 2016	EPIC-Spain	X	X	X

ARIC, indicates Atherosclerosis Risk in Communities Study; EPIC-Spain, European Prospective Investigation into Cancer and Nutrition-Spain; HPFS, Health Professionals Follow-Up Study; NHS, Nurses' Health Study.

[a]X, indicates primary study included in systematic review.

Table S12. Assessment for the different items of AMSTAR 2 and rationale behind assessment for the critical domains of AMSTAR 2

Assessment for the different items of AMSTAR 2

AMSTAR 2 items[a]	Systematic review		
	Kim 2017	Bechthold 2019	Zeraatkar 2019
Item 1 Did the research questions and inclusion criteria for the review include the components of Population, Intervention, Comparator, Outcome (PICO)?	Yes	Yes	Yes
Item 2 Did the report of the review contain an explicit statement that the review methods were established prior to the conduct of the review and did the report justify any significant deviations from the protocol?	No	Yes	Partial yes
Item 3 Did the review authors explain their selection of the study designs for inclusion in the review?	Yes	Yes	Yes
Item 4 Did the review authors use a comprehensive literature search strategy?[b]	Yes	Yes	Partial yes
Item 5 Did the review authors perform study selection in duplicate?	Yes	Yes	Yes
Item 6 Did the review authors perform data extraction in duplicate?	Yes	No	Yes
Item 7 Did the review authors provide a list of excluded studies and justify the exclusions?	No	Yes	No
Item 8 Did the review authors describe the included studies in adequate detail?	Partial yes	Partial yes	Partial yes
Item 9 Did the review authors use a satisfactory technique for assessing the risk of bias in primary studies that were included in the review?[c]	Yes	No	Yes
Item 10 Did the review authors report on the sources of funding for the studies included in the review?	No	No	Yes
Item 11 If meta-analysis was performed did the review authors use appropriate methods for statistical combination of results?[d]	Yes	Yes	Yes
Item 12 If meta-analysis was performed, did the review authors assess the potential impact of risk of bias in primary studies on the results of the meta-analysis or other evidence synthesis?[d]	No	Yes	Yes
Item 13 Did the review authors account for risk of bias in primary studies when interpreting/discussing the results of the review?	Yes	Yes	Yes
Item 14 Did the review authors provide a satisfactory explanation for, and discussion of, any heterogeneity observed in the results of the review?	Yes	Yes	No
Item 15 If they performed quantitative synthesis did the review authors carry out an adequate investigation of publication bias (small study bias) and discuss its likely impact on the results of the review?[d]	Yes	No	No
Item 16 Did the review authors report any potential sources of conflict of interest, including any funding they received for conducting the review?	Yes[e]	Yes[e]	Yes[f]

[a]The domain-specific questions are framed so that a 'Yes' answer denotes a positive result. All steps in the conduct of a systematic review and meta-analysis are important, but seven domains can critically affect the validity of a review and its conclusions. These domains are highlighted with orange.

[b]We decided not to include 'searched study registries' in our assessment. This was because most cohort studies (contemporary studies) are not registered before being conducted. Furthermore, 'included/consulted content experts in the field' was not considered relevant because only a biased sample of such studies can be identified and because unpublished studies may tend to be of lower quality. Also, we did not include 'searched for grey literature' as grey literature may not have been subject to peer review and therefore may be of lower quality.

[c]We decided not to include 'selection of the reported result from among multiple measurements or analyses of a specified outcome' in our assessment. This was because most cohort studies are not registered before being conducted.

[d]If a high versus low (or a low versus high) intake meta-analysis as well as a dose-response meta-analysis was available for an exposure, we assessed the dose-response meta-analysis.

[e]The authors reported no competing interests.

[f]The authors described their funding sources and how they managed potential conflicts of interest.

Rationale behind assessment for the critical domains of AMSTAR 2

The AMSTAR 2 [1] appraisal tool was used to assess the quality of how each included systematic review was conducted and to rate the overall confidence in the results of each review, as detailed in Supplementary Materials Methods S2. All steps in the conduct of a systematic review and meta-analysis are important, but seven domains (items 2, 4, 7, 9, 11, 13, and 15) can critically affect the validity of a review and its conclusions [1]. The rationale behind partial positive results ('Partial yes' answers) and negative results ('No' answers) of the assessment for the critical domains is given below.

Unprocessed red meat

One systematic review [2] investigated the association between unprocessed red meat and cardiovascular disease (CVD), and two systematic reviews [2,3] investigated the association between unprocessed red meat and stroke. Each review was rated as critically low, which means that 'the systematic review has more than one critical flaw and should not be relied on to provide an accurate and comprehensive summary of the available studies' [1].

Zeraatkar et al. [2] stated that they had a written protocol but deviations (in review question and outcome) from the protocol were not justified (item 2, 'Partial yes'). They searched six databases, provided search strategy, and conducted search within 24 months of completion of the review but they did not search the reference lists of included studies (item 4, 'Partial yes'). Furthermore, they did not use graphical display or statistical tests to detect publication bias and they did not discuss the likelihood and magnitude of impact of publication bias (item 15, 'No'). Kim et al. [3] did not state that they had a written protocol or guide (item 2, 'No'). In none of the reviews [2,3] was a list provided of all potentially relevant studies that were retrieved and assessed for eligibility but excluded from the review (item 7, 'No').

Unprocessed poultry

One systematic review [3] investigated the association between unprocessed poultry and stroke. The review was rated as critically low, which means that 'the systematic review has more than one critical flaw and should not be relied on to provide an accurate and comprehensive summary of the available studies' [1].

The authors did not state that they had a written protocol or guide (item 2, 'No'), and a list of all potentially relevant studies that were retrieved and assessed for eligibility but excluded from the review was not provided (item 7, 'No') [3].

Processed meat

One systematic review [2] investigated the association between processed meat and CVD, one systematic review [4] investigated the association between processed meat and coronary heart disease, and three systematic reviews [2–4] investigated the association between processed meat and stroke. Each review was rated as critically low, which means that 'the systematic review has more than one critical flaw and should not be relied on to provide an accurate and comprehensive summary of the available studies' [1].

Zeraatkar et al. [2] stated that they had a written protocol but deviations (in review question and outcome) from the protocol were not justified (item 2, 'Partial yes'). They searched six databases, provided search strategy, and conducted search within 24 months of completion of the review but they did not search the reference lists of included studies (item 4, 'Partial yes'). Furthermore, a list of all potentially relevant studies that were retrieved and assessed for eligibility but excluded from the review was not provided (item 7, 'No') and they did not use graphical display or statistical tests to detect publication bias and they did not discuss the likelihood and magnitude of impact of publication bias (item 15, 'No'). Bechthold et al. [4] assessed the risk of bias from confounding and from methods used to ascertain exposures and outcomes in primary studies but not from selection bias (item 9, 'No'). Furthermore, they did not use graphical display or statistical tests to detect publication bias and they did not discuss the likelihood and magnitude of impact of publication bias (item 15, 'No'). Kim et al. [3] did not state that they had a written protocol or guide (item 2, 'No') and a list of all potentially relevant studies that were retrieved and assessed for eligibility but excluded from the review was not provided (item 7, 'No').

References

1. Shea, B.J.; Reeves, B.C.; Wells, G.; Thuku, M.; Hamel, C.; Moran, J.; Moher, D.; Tugwell, P.; Welch, V.; Kristjansson, E.; et al. AMSTAR 2: A Critical Appraisal Tool for Systematic Reviews That Include Randomised or Non-Randomised Studies of Healthcare Interventions, or Both. *BMJ* **2017**, *358*, j4008, doi:10.1136/bmj.j4008.

2. Zeraatkar, D.; Han, M.A.; Guyatt, G.H.; Vernooij, R.W.M.; Dib, R. El; Cheung, K.; Milio, K.; Zworth, M.; Bartoszko, J.J.; Valli, C.; et al. Red and Processed Meat Consumption and Risk for All-Cause Mortality and Cardiometabolic Outcomes: A Systematic Review and Meta-Analysis of Cohort Ctudies. *Ann. Intern. Med.* **2019**, *171*, 703–710, doi:10.7326/M19-0655.

3. Kim, K.; Hyeon, J.; Lee, S.A.; Kwon, S.O.; Lee, H.; Keum, N.; Lee, J.; Park, S.M. Role of Total, Red, Processed, and White Meat Consumption in Stroke Incidence and Mortality: A Systematic Review and Meta-Analysis of Prospective Cohort Studies. *J. Am. Heart Assoc.* **2017**, *6*, e005983, doi:10.1161/JAHA.117.005983.

4. Bechthold, A.; Boeing, H.; Schwedhelm, C.; Hoffmann, G.; Knüppel, S.; Iqbal, K.; De Henauw, S.; Michels, N.; Devleesschauwer, B.; Schlesinger, S.; et al. Food Groups and Risk of Coronary Heart Disease, Stroke and Heart Failure: A Systematic Review and Dose-Response Meta-Analysis of Prospective Studies.

Crit. Rev. Food Sci. Nutr. **2019**, *59*, 1071–1090, doi:10.1080/10408398.2017.1392288.

Table S13. Scoring for the different items of NutriGrade (maximum of 10 points) for primary study meta-analyses of unprocessed red meat

First author's last name and publication year	Outcome	Meta-analysis	Item 1 Risk of bias in individual cohort studies (0-2 points)	Item 2 Precision of the estimate (0-1 points)	Item 3 Heterogeneity (0-1 points)	Item 4 Directness (0-1 points)	Item 5 Publication bias (0-1 points)	Item 6 Funding bias (0-1 points)	Item 7 Effect size (0-2 points)	Item 8 Dose-response association (0-1 points)	NutriGrade score	NutriGrade grading of quality of evidence[a]
Zeraatkar 2019	CVD	Dose-response	0.0	1.0	0.0	1.0	0.0	1.0	0.0	0.0	3.0	Very low[b]
Kim 2017	Stroke	High versus low	2.0	1.0	0.4	1.0	0.5	1.0	0.0	0.0	5.9	Low[c]
Zeraatkar 2019	Stroke	Dose-response	2.0	1.0	0.4	1.0	0.0	1.0	0.0	1.0	6.4	Moderate[d]

CVD, indicates cardiovascular disease.

[a]Four categories are established to grade the quality of evidence of meta-analyses. A score of ≥8 points is assigned to high, 6 to <8 points to moderate, 4 to <6 points to low, and <4 points to very low. In Zeraatkar 2019, scoring for effects size was based on summary risk ratio estimates from dose-response meta-analysis.

[b]There is very low confidence in the effect estimate; meta-evidence is very limited and uncertain.

[c]There is low confidence in the effect estimate; further research will provide important evidence on the confidence and likely change the effect estimate.

[d]There is moderate confidence in the effect estimate; further research could add evidence on the confidence and may change the effect estimate.

Table S14. Scoring for the different items of NutriGrade (maximum of 10 points) for primary study meta-analyses of unprocessed poultry

First author's last name and publication year	Outcome	Meta-analysis	Item 1 Risk of bias in individual cohort studies (0-2 points)	Item 2 Precision of the estimate (0-1 points)	Item 3 Heterogeneity (0-1 points)	Item 4 Directness (0-1 points)	Item 5 Publication bias (0-1 points)	Item 6 Funding bias (0-1 points)	Item 7 Effect size (0-2 points)	Item 8 Dose-response association (0-1 points)	NutriGrade score	NutriGrade grading of quality of evidence[a]
Kim 2017	Stroke	High versus low[b]	2.0	1.0	0.0	1.0	0.0	1.0	0.0	0.0	5.0	Low[b]

[a]Four categories are established to grade the quality of evidence of meta-analyses. A score of ≥8 points is assigned to high, 6 to <8 points to moderate, 4 to <6 points to low, and <4 points to very low.

[b]There is low confidence in the effect estimate; further research will provide important evidence on the confidence and likely change the effect estimate.

Table S15. Scoring for the different items of NutriGrade (maximum of 10 points) for primary study meta-analyses of processed meat

First author's last name and publication year	Outcome	Meta-analysis	Item 1 Risk of bias in individual cohort studies (0-2 points)	Item 2 Precision of the estimate (0-1 points)	Item 3 Heterogeneity (0-1 points)	Item 4 Directness (0-1 points)	Item 5 Publication bias (0-1 points)	Item 6 Funding bias (0-1 points)	Item 7 Effect size (0-2 points)	Item 8 Dose-response association (0-1 points)	NutriGrade score	NutriGrade grading of quality of evidence[a]
Zeraatkar 2019	CVD	Dose-response	0.0	1.0	0.0	1.0	0.0	1.0	0.0	0.0	3.0	Very low[b]
Bechthold 2019	CHD	Dose-response	n/a	n/a	n/a	n/a	n/a	n/a	n/a	n/a	n/a	Moderate[c,d]
Kim 2017	Stroke	High versus low	2.0	1.0	0.4	1.0	0.5	1.0	0.0	0.0	5.9	Low[e]
Bechthold 2019	Stroke	Dose-response	n/a	n/a	n/a	n/a	n/a	n/a	n/a	n/a	n/a	Moderate[c,d]
Zeraatkar 2019	Stroke	Dose-response	2.0	1.0	0.2	1.0	0.0	1.0	0.0	1.0	6.2	Moderate[c]

CHD, indicates coronary heart disease; CVD, cardiovascular disease; n/a, not provided, because the answer is not available from the systematic review.

[a]Four categories are established to grade the quality of evidence of meta-analyses. A score of ≥8 points is assigned to high, 6 to <8 points to moderate, 4 to <6 points to low, and <4 points to very low. In Zeraatkar 2019, scoring for effects size was based on summary risk ratio estimates from dose-response meta-analysis.

[b]There is very low confidence in the effect estimate; meta-evidence is very limited and uncertain.

[c]There is moderate confidence in the effect estimate; further research could add evidence on the confidence and may change the effect estimate.

[d]Systematic review authors' grading. The scoring for the different items is not reported.

[e]There is low confidence in the effect estimate; further research will provide important evidence on the confidence and likely change the effect estimate.

Table S16. High versus low meta-analyses of associations between intake of unprocessed red meat and risk of CVD and stroke

First author's last name and publication year	Outcome	Number of studies	Summary risk ratio (95% CI)	Heterogenity (I^2)
Zeraatkar 2019	CVD	4	1.09 (0.94, 1.25)[a]	22.7%
Kim 2017	Stroke	6	1.11 (1.03, 1.20)	0.0%
Zeraatkar 2019	Stroke	6	1.11 (1.03, 1.20)[a]	0.1%

CI, indicates confidence interval; CVD, cardiovascular disease.

[a]Risk ratio converted from low versus high intake to high versus low intake by using the formula $RR_{high\ vs.\ low} = 1/RR_{low\ vs.\ high}$.

Table S17. High versus low meta-analyses of associations between intake of unprocessed poultry and risk of stroke

First author's last name and publication year	Outcome	Number of studies	Summary risk ratio (95% CI)	Heterogenity (I^2)
Kim 2017	Stroke	3	0.87 (0.78, 0.96)	0.0%

CI, indicates confidence interval.

Table S18. High versus low meta-analyses of associations between intake of processed meat and risk of CVD, CHD, and stroke

First author's last name and publication year	Outcome	Number of studies	Summary risk ratio (95% CI)	Heterogenity (I^2)
Zeraatkar 2019	CVD	4	1.03 (0.95, 1.14)[a]	0.0%
Bechthold 2019	CHD	5	1.15 (0.99, 1.33)	44%
Kim 2017	Stroke	6	1.17 (1.08, 1.25)	0.0%
Bechthold 2019	Stroke	6	1.16 (1.07, 1.26)	12%
Zeraatkar 2019	Stroke	6	1.18 (1.08, 1.25)[a]	0.0%

CHD, indicates coronary heart disease; CI, confidence interval; CVD, cardiovascular disease.

[a]Risk ratio converted from low versus high intake to high versus low intake by using the formula $RR_{high\ vs.\ low} = 1/RR_{low\ vs.\ high}$.
